# Healthcare Workers’ Worries and Monkeypox Vaccine Advocacy during the First Month of the WHO Monkeypox Alert: Cross-Sectional Survey in Saudi Arabia

**DOI:** 10.3390/vaccines10091408

**Published:** 2022-08-28

**Authors:** Fadi Ajman, Shuliweeh Alenezi, Khalid Alhasan, Basema Saddik, Ali Alhaboob, Esraa S. Altawil, Fatimah Alshahrani, Abdulkarim Alrabiaah, Ali Alaraj, Khaled Alkriadees, Yousef Alshamlani, Homood Alharbi, Amr Jamal, Rabih Halwani, Fahad AlZamil, Sarah Al-Subaie, Mazin Barry, Ziad A. Memish, Jaffar A. Al-Tawfiq, Mohamad-Hani Temsah

**Affiliations:** 1 College of Medicine, King Saud University, Riyadh 11362, Saudi Arabia; 2 Critical Care Department, King Saud University Medical City, King Saud University, Riyadh 11362, Saudi Arabia; 3 Department of Psychiatry, College of Medicine, King Saud University Medical City, King Saud University, Riyadh 11362, Saudi Arabia; 4 Pediatric Department, King Saud University Medical City, King Saud University, Riyadh 11362, Saudi Arabia; 5 Pediatric Kidney Transplant Section, Organ Transplant Center of Excellence, King Faisal Specialist Hospital and Research Center, Riyadh 11411, Saudi Arabia; 6 Department of Family and Community Medicine, College of Medicine, University of Sharjah, Sharjah 27272, United Arab Emirates; 7 Sharjah Institute of Medical Research, College of Medicine, University of Sharjah, Sharjah 27272, United Arab Emirates; 8 Clinical Pharmacy Services, Pharmacy Department, King Saud University Medical City, Riyadh 11362, Saudi Arabia; 9 Division of Infectious Diseases, Department of Internal Medicine, King Saud University Medical City, King Saud University, Riyadh 11362, Saudi Arabia; 10 Department of Medicine, Qassim University, Qassim 51452, Saudi Arabia; 11 Department of Medicine, Al-Habib Hospital, Riyadh 12214, Saudi Arabia; 12 Department of Family and Community Medicine, King Saud University Medical City, Riyadh 11362, Saudi Arabia; 13 Department of Clinical Sciences, College of Medicine, University of Sharjah, Sharjah 27272, United Arab Emirates; 14 Division of Infectious Diseases, Faculty of Medicine, University of Ottawa, Ottawa, ON K1H 8M5, Canada; 15 Research and Innovation Center, King Saud Medical City, Ministry of Health & College of Medicine, Alfaisal University, Riyadh 11533, Saudi Arabia; 16 Hubert Department of Global Health, Emory University, Atlanta, GA 30322, USA; 17 Specialty Internal Medicine and Quality Department, Johns Hopkins Aramco Healthcare, Dhahran 34465, Saudi Arabia; 18 Infectious Disease Division, Department of Medicine, Indiana University School of Medicine, Indianapolis, IN 46202, USA; 19 Infectious Disease Division, Department of Medicine, Johns Hopkins University School of Medicine, Baltimore, MD 21218, USA; 20 Prince Abdullah bin Khaled Coeliac Disease Research Chair, King Saud University, Riyadh 11362, Saudi Arabia

**Keywords:** Monkeypox versus COVID-19, HCWs’ perceptions, Monkeypox worries, Monkeypox vaccine acceptance, HCWs in Saudi Arabia

## Abstract

**Background:** Monkeypox virus re-surged in May 2022 as a new potential global health threat, with outbreaks bursting in multiple countries across different continents. This study was conducted in Saudi Arabia during the first month following the WHO announcement of the Monkeypox outbreak, to assess healthcare workers (HCWs) perceptions of, worries concerning, and vaccine acceptance for, Monkeypox, in light of the resolving COVID-19 pandemic. **Methods:** A national cross-sectional survey was conducted between 27 May and 10 June 2022, in Saudi Arabia. Data were collected on: HCWs’ sociodemographic and job-related characteristics; COVID-19 infection status; and worries concerning Monkeypox, compared to COVID-19 and its sources; as well as their perceptions and awareness of, and advocacy for, supporting Monkeypox vaccination. **Results:** A total of 1130 HCWs completed the survey, of which 41.6% have already developed COVID-19. However, 56.5% were more concerned about COVID-19 compared to Monkeypox, while the rest were more worried about Monkeypox disease. The main cause for concern among 68.8% of the participants was the development of another worldwide pandemic, post-COVID-19, followed by their concern of either themselves or their families contracting the infection (49.6%). Most HCWs (60%) rated their level of self-awareness of Monkeypox disease as moderate to high. Males, and those who had previously developed COVID-19, were significantly less likely to worry about Monkeypox. The worry about Monkeypox developing into a pandemic, and the perception of Monkeypox being a severe disease, correlated significantly positively with the odds of high worry concerning the disease. The major predictors of participants’ advocacy for vaccination against Monkeypox disease were: those who had developed COVID-19 previously; and those who supported tighter infection control measures (than those currently used) to combat the disease. A total of 74.2% of the surveyed HCWs perceived that they needed to read more about Monkeypox disease. **Conclusions:** Approximately half of the HCWs in this study were more concerned about Monkeypox disease than COVID-19, particularly regarding its possible progression into a new pandemic, during the first month following the WHO’s Monkeypox international alert. In addition, the majority of participants were in favor of applying tighter infection prevention measures to combat the disease. The current study highlights areas requiring attention for healthcare administrators regarding HCWs’ perceptions and preparedness for Monkeypox, especially in the event of a local or international pandemic.

## 1. Introduction

The recent reemergence of the Monkeypox virus (MPXV) has raised considerable concerns regarding the possibility of developing another pandemic in the shadow of COVID-19. MPXV is zoonotic in origin, with multiple reservoirs. MPXV refers to the first isolation from captive monkeys shipped to the Netherlands from Africa in 1958 which was later identified (for the first time) in humans, in 1970 [1]. The recent multi-country outbreak of MPXV, without travel to endemic areas, is certainly a cause for concern [1,2]. This concern was exemplified by scientists around the globe [3]. The World Health Organization (WHO) called for an international meeting to discuss the importance of MPXV, which concluded that the event did not constitute a public health emergency of international concern (PHEIC). However, the emergence of MPXV continues to add to the current burden of anxiety experienced by healthcare workers (HCWs) and the public [4]. A recent study revealed that approximately 62% of the general population were more concerned about MPXV than COVID-19 [5].

The ongoing COVID-19 pandemic has been associated with stress among HCWs, as well as increased workload and anxiety [6]. One of the approaches used to control the current outbreak of MPXV is vaccination. Such a strategy is being utilized in certain countries, such as the US, Canada, and Europe [7]. However, such a strategy is associated with challenges and unknowns [7]. Among the 521 cases of MPXV reported in Germany, the median age was 38 years, and all cases were men [8]. Similarly, the reported cases from Spain indicated a predominance of men who had sex with men [9]. Other studies have examined the global estimates of MPXV cases across several nations [10,11]. However, the experience from the COVID-19 pandemic has enabled many countries worldwide to develop rapid testing, isolation, and management [10]. Additionally, some countries have deployed research electronic data capture (REDCap) design and programmers, as well as infection-control activities, in response to MPXV outbreaks, globally [12]. Amid these unknowns, and the need to develop a global strategy to combat the emergence of MPXV, we rapidly developed a survey to assess the worries and concerns among HCWs in Saudi Arabia, and their advocacy of the Monkeypox vaccine during the first month of the WHO Monkeypox alert.

## 2. Method

### 2.1. Data Collection

An online survey of healthcare workers in the Kingdom of Saudi Arabia (KSA) was conducted over ten days (from 27 May–5 June 2022). Participants were invited, by convenience sampling techniques, through various social media platforms (Twitter and WhatsApp groups) and email lists. Participants were invited to complete the online survey through the SurveyMonkey© platform, with each response allowed once from each unique IP address, to ensure single entries. The first page of the survey included consent of participation, explained the study research objectives, and assured confidentiality.

The survey tool was adopted from our published research on COVID-19, with modifications relating to the new Monkeypox outbreak [13,14,15,16,17]. The final version was piloted among ten HCWs for clarity and consistency. Modifications were implemented based on the experts’ recommendations. The questionnaire took eight minutes to complete. The research team approved the final version of the survey for language accuracy, clarity, and content validity.

Variables surveyed included: HCWs’ sociodemographic and job-related characteristics; worries concerning Monkeypox compared to COVID-19; sources of worries; previous COVID-19 infection status; and advocacy for Monkeypox disease vaccination. Also, we assessed their compliance with infection prevention precautionary measures against COVID-19, and their support for tighter measures regarding the MPXV outbreaks. Finally, their generalized anxiety disorder (GAD7) score [18,19]—which is a self-reported, 7-item validated scale—was used as a measure of anxiety.

Assuming 50% of the sample will demonstrate sufficient knowledge about Monkeypox, the minimum desired sample size required to detect the true proportion of participants, with the outcome of 95% confidence and a margin of error of 5%, was estimated to be equal to 384 subjects; however, the achieved sample size was 1130.

### 2.2. Ethical Approval

Ethical approval was granted by the institutional review board (IRB) at King Saud University (22/0416/IRB) before data collection began.

### 2.3. Statistical Analysis

Means and standard deviations were used to describe continuous variables, frequencies and percentages for categorically measured variables. The histogram and the Kolmogorov-Smirnov test were applied to test the assumption of normality, and Levene’s test was used to test the homogeneity of variance statistical assumption. Cronbach’s alpha test was used to assess the internal consistency of the measured questionnaires. The Multivariate binary logistic regression analysis was used to assess variables independently correlating with HCWs’ worry regarding MPXV, their advocacy for, or against, HCWs’ vaccination, and their perception of the need to apply tighter infection prevention practices (IPC) to combat the MPXV outbreaks. The association between predictors with the outcome-dependent variables in the multivariate logistic binary regression analysis was expressed with adjusted odds ratio (OR), with their associated 95% confidence intervals. The SPSS IBM statistical analysis program was used for statistical data analysis. The statistical alpha significance level was considered at 0.050 level.

## 3. Results

A total of 1130 HCWs residing in Saudi Arabia participated in the study. Table 1 displays the sociodemographic and professional characteristics. The mean age was (37.1, SD = 9.69 years). Of the participants, 62.7% were females, 66.2% were married, and 57% were expatriate.

Of the respondents, 62.1% were working in tertiary institutions, while the rest were divided equally between primary and secondary centers. Regarding their clinical roles, 42.7% were nurses, 39.1% were physicians, and their clinical assignment area was distributed across the different departments of the healthcare institutions.

41.6% of participants reported having had COVID-19.

Table 2 details the HCWs’ awareness and sources of information about Monkeypox disease. About half of them traveled to countries that recently reported Monkeypox disease, mainly Europe, North America, Australia (52.4%), and the UAE (23.3%), while only (5.8%) reported traveling to Western or Central Africa. A total of 60% of the surveyed HCWs’ rated their awareness about the Monkeypox disease, at the time of the study, as moderate to high. Interestingly, 74.2% of the surveyed HCWs perceived that they needed to read more about the Monkeypox disease after receiving the current survey.

HCWs’ Monkeypox disease perceptions and worries are displayed in Table 3. Their self-rated worry of the Monkeypox disease developing into a worldwide pandemic ranged from none/a little worried (48.7%), moderate (26%), to ‘worried a lot’ (25.3). Considering the current Monkeypox outbreak, 20.4% of HCWs perceive no worry at all about travelling abroad, 66.1% were somewhat worried, and 13.5% were extremely worried. The main sources of worry for respondents were concerning: themselves or their families acquiring the infection (49.6%); the development of another worldwide pandemic (68.8%); a national lockdown (42.6%); and international travel restrictions (40.8%). When asked whether Monkeypox disease can cause more severe symptoms when compared to smallpox disease, 23.4% disagreed, while the rest were split equally between agree or unsure.

Currently, 56.5% of HCWs are more worried about COVID-19 than Monkeypox, 35.7% are more worried about Monkeypox, while 7.8% of HCWs are unsure or equally worried about both diseases. Regarding the need to implement more infection control measures than those currently in place, 67.6% of respondents agreed, 25.9% were unsure and 6.5% disagreed.

**Figure 1:**
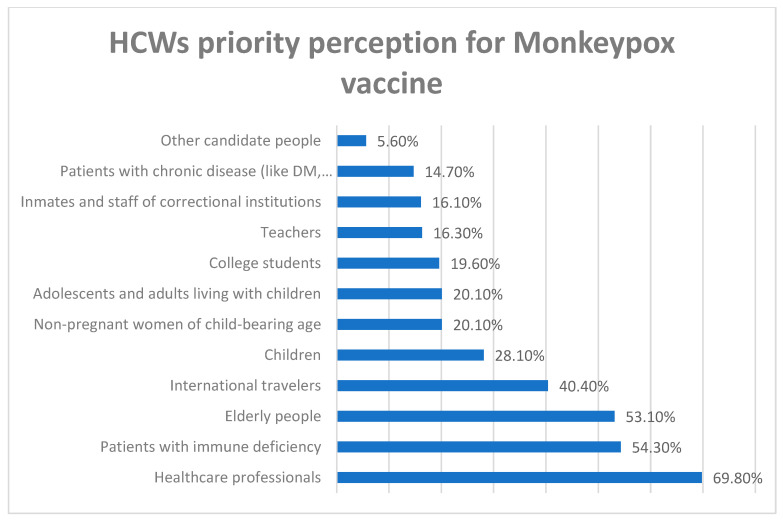
HCWs’ priority perception for Monkeypox vaccine.

Figure 1 illustrates the details of HCWs’ prioritization of administering the Monkeypox vaccine. Most (69.8%) HCWs believed they should be vaccinated first, followed by patients with immune deficiency (54.3%), the elderly (53.1%), then international travelers (40.4%).

We assessed variables associated with HCWs’ odds of having high worry from Monkeypox, using multivariate binary logistic regression analysis (Table 4). Compared to females, males were significantly less likely to be concerned about Monkeypox (OR = 0.533, *p* < 0.001), while HCWs’ age, household size, and GAD7 score did not correlate significantly with their level of worry from Monkeypox disease. Those who had previously developed COVID-19 were significantly less worried (OR = 0.735, *p* = 0.026). With regard to the participants’ clinical roles, medical students were significantly more worried, compared to the other surveyed participants (OR 2.79, *p* < 0.001). HCWs’ self-rated awareness level of the Monkeypox disease correlated significantly, but negatively, with their odds of high worry from Monkeypox disease (OR = 0.765, *p* < 0.001). The worries among HCWs of Monkeypox progressing into a pandemic correlated significantly, and positively, with their odds of high worry from the disease (OR = 1.465 *p* < 0.001), in addition to their perception of Monkeypox as a severe disease (OR = 1.26, *p* = 0.017) and those who agreed with implementing tighter infection control measures than current measures in place to control the disease (OR = 1.691, *p* < 0.001). However, HCWs’ worry about international travel restrictions did not correlate significantly with their odds of high worry from the Monkeypox disease *p* = 0.151.

Table 5 presents a multivariate binary logistic regression analysis of the odds of HCWs agreeing to be vaccinated against Monkeypox in the current stage of the outbreak. HCWs’ sex, age, marital status, and healthcare institution type did not correlate significantly with their odds of agreement with vaccination. While those who had previously developed COVID-19 were significantly more likely to agree (OR = 1.327, *p*-value = 0.043). Additionally, HCWs who supported the application of tighter infection control measures to combat Monkeypox disease were significantly more likely to support vaccination (OR = 1.415, *p* = 0.003). HCWs’ perception of needing to read more about the disease and their GAD7 score did not correlate significantly with their likelihood of supporting vaccination. The sources of information used by HCWs correlated significantly and positively with their odds of supporting vaccination but with variable strength, from those who relied on local official sources (OR = 2.023, *p* < 0.001) to social networks (OR = 1.953, *p* < 0.001) and international health websites (OR = 1.375, *p* < 0.001).

To further assess HCWs’ perceptions regarding the Monkeypox disease and their worries, we conducted a multivariate binary logistic regression analysis, to identify the variables that are independently associated with their support for tighter infection control measures against Monkeypox disease, compared to the ones currently adopted after the COVID-19 pandemic (Table 6). HCWs’ sex, age, marital status, clinical role, and GAD7 score did not correlate significantly with their likelihood to support tighter measures. However, their perceived worry from the severity of the Monkeypox disease correlated significantly and positively with their odds of supporting the application of tighter infection control measures against the Monkeypox disease (OR = 1.316, *p* = 0.009). This was also reflected in their perception of the need to read more about Monkeypox, which was significantly and positively associated with support for the application of tighter measures (OR = 3.157, *p* < 0.001). In terms of their sources of information about Monkeypox disease, those who used social media networks were more likely to support tighter infection control measures (OR = 1.35, *p* = 0.046). In comparison, those who relied primarily on scientific journals were less likely to support tighter infection control measures (OR = 0.656, *p* = 0.023). Those who relied on international health websites did not have any convergence with their odds to support tighter control measures. HCWs who were more worried about Monkeypox, compared to COVID-19, were significantly more likely to support tighter infection control measures (OR = 2.067, *p* < 0.001). HCWs who were concerned about international travel restrictions had higher support for the implementation of tighter measures (OR = 1.568, *p* = 0.002). In comparison, those who were mainly worried about the Monkeypox outbreak progressing into a pandemic had significantly lower support for applying tighter measures (OR = 0.813, *p* = 0.006). HCWs who lived with more than three people were significantly more likely to support tighter infection control measures than those living with fewer than three people (OR = 1.230, *p* = 0.021).

## 4. Discussion

The international health care system experienced one of the largest and most stressful pandemics in recent decades, namely COVID-19, which caused a tremendous burden. This was related to expansion demand within a very short time, and high anxiety and worry about acquiring the disease. With the recovery of the healthcare system after those two years of the pandemic, and massive vaccination campaigns that targeted not only medical practitioners but also the public, this caused great demand, requiring extra effort within the healthcare system. The re-emergence of the Monkeypox disease just within the resolution phase of the COVID-19 pandemic has been a challenging alert for the health care system, especially with having lost the applied infection prevention measures introduced during the COVID-19 pandemic. The Monkeypox disease was described for the first time in 1970 in the Democratic Republic of the Congo [20]. Since then, multiple cases have been described, mainly in African countries, with occasional outbreaks, as in 1996–1997 in the Democratic Republic of the Congo, and in Nigeria in 2017 [20]. However, it has been described outside Africa since 2003. The recent re-emergence of Monkeypox, in outbreak fashion, in multiple countries simultaneously, and its relation to sexual activity and intimate body interaction, such as: men having sex with men (MSM); participating in extended sexual networks; plus the multiple modes of transmission of the disease, involving respiratory droplets, skin lesions, and sexual contact; all heightened the alert within the medical system [21].

Our study has shown that 56.5% of the HCWs were more worried about COVID-19 when compared to Monkeypox; 48.7% had no to little worry about its progression into a worldwide pandemic, while 25.3% were worried a lot. A total of 67.6% agreed with applying tighter infection control measures (compared to those currently applied) in order to combat the current outbreak of Monkeypox disease. The worry that Monkeypox could evolve into a pandemic was described in the literature, both before and after the inception of the pandemic [22,23,24]. Monkeypox does not readily transmit from person to person, and because it is related to the smallpox virus, there are treatments and vaccines already available. So, although scientists worldwide are concerned about such new viral behavior, they are not panicked [25].

Furthermore, HCWs’ self-rated awareness level of the Monkeypox disease, expectedly, correlated significantly, but negatively, with their odds of high worry from Monkeypox disease. Similarly, the HCWs’ worry of Monkeypox progressing into a pandemic correlates with their perceived severity of the Monkeypox disease, and agreement with tighter infection control measures.

Male HCWs in Saudi Arabia were less predicted to worry about Monkeypox than females, which is a common observation of gender difference in relation to infectious disease risks such as COVID-19 in HCWs [26,27]. This difference, interestingly, resulted in a huge mental health burden and impact on the workforce [28]. However, the participants’ clinical role showed that medical students were significantly more worried compared to the other surveyed participants. This finding was not surprising, as the COVID-19 pandemic affected medical students more, and even raised their concerns to reconsider their profession [29]. HCWs who previously developed COVID-19 were also significantly less worried about the Monkeypox disease outbreak, which is probably related to having already survived through the COVID-19 pandemic and even the infection itself; some reports have shown shorter recovery time from COVID-19 and milder symptoms in HCWs, and their medical knowledge background of the Monkeypox disease, with its limited potential of transmission compared to COVID-19, mostly led to lower worry levels in HCWs who were COVID-19 survivors [30]. In line with the HCWs’ medical knowledge background about the Monkeypox disease, its vulnerability, and their ability to critically interpret the transmission facts about its most recent outbreak, their self-rated awareness of the disease correlated negatively with their odds of higher worry from the disease. In contrast, HCWs who had a high concern of Monkeypox progression into a pandemic, and those who perceived the disease as potentially being severe, had higher worry. Similar findings were also observed for those who potentially perceived the disease dangerous; despite the scientific background basis of their perception; similar observation has been described in HCWs in contagion with COVID-19 patients [31,32]. In line with the previous observation, HCWs who perceived the need to apply tighter infection control measures (than those currently applied to limit Monkeypox progress) also had odds of high worry from the disease. Such observations of HCWs’ perceptions of Monkeypox disease, its severity, and how to combat it, are similar to COVID-19 studies and might reflect the anxious temperament of the respondents [33].

HCWs’ perceptions in relation to MPXV, according to our results, have shown that 48.7% had none/little worry from MPXV to progress to a pandemic, while only 25.3% were worried a lot. A total of 35.7% were more worried of MPXV, in comparison to COVID-19, and only 36.8% perceived or agreed that MPXV can cause more severe disease compared to smallpox. Therefore, in conclusion, our participants didn’t have largely major concerns of MPXV. Another major point to keep in mind is the societal response to emerging infectious disease, especially in the acute stage, when a lot of unknowns are there, usually society handles such threats with fear, panic, and calls-of-action in a mode of “all in war against all”; adding to that any residual concerns and experiences the HCWs passed through during the COVID-19 pandemic may put them on the edge sometimes, even without scientific rationale [34].

Currently, there are two vaccines that are being suggested against Monkeypox. ACAM2000 is a second-generation smallpox vaccine made from vaccinia virus, which has activity against Monkeypox [35], and JYNNEOS vaccine (also known as Imvanex, or Imvamune) is a live non-replicating vaccine produced from a modified strain of Vaccinia Ankara-Bavarian Nordic (MVA-BN) orthopoxvirus, which has previously been given to HCWs who were involved in investigating Monkeypox outbreaks [36]. Advocacy for vaccinating HCWs against Monkeypox deserves further studies in the context of the reemergence of the disease and recent outbreaks. The current study did not find any correlation between HCWs’ different demographics and vaccine acceptance. However, those who had been previously infected with COVID-19 were more likely to support vaccination against Monkeypox. A previous study from KSA, which examined factors related to COVID-19 vaccine acceptance among HCWs before its availability, did not show higher acceptance among individuals previously infected with COVID-19, but after two years of the pandemic and experiencing the protection effect of vaccination campaigns, HCWs probably felt more encouraged toward Monkeypox vaccination, with increasing confidence in vaccination in general; this was observed with Flu vaccine during the COVID-19 pandemic, and vaccination in general [37,38,39]. Interestingly, HCWs who advocated for tighter infection prevention and control measures were also more supportive of vaccination, both in line with their healthy behavior and their belief that both are integral to each other; this echoes a previous study that showed a higher rate of hepatitis B vaccination in HCWs who attended infection control training [40]. Other possible infection prevention and control measures applicable, if the outbreak evolves further, may include pre-exposure prophylaxis with vaccines [41]. This seems to be welcomed by the surveyed HCWs. Furthermore, we identified that HCWs in KSA who rely on official local statements from Saudi MOH and CDC were more likely to accept vaccination. Reliance on the latter sources has been previously proven to increase COVID-19 vaccine acceptance in numerous studies from KSA; this study further supports utilizing these sources for accurate and reliable information [15,17,42,43].

In this study, there was a significant correlation between the high level of worry concerning Monkeypox disease and the need to expand HCWs’ one-sided information about the disease, especially with regard to supporting tighter infection control measures. Interestingly, relying on the medical and scientific journals was not associated with HCWs’ belief in tighter infection control measures to combat the outbreak; this was in contradiction to those who relied on social media. That is in line with previous studies of the COVID-19 pandemic among HCWs which showed that the top three sources of HCWs for information were social media, the WHO website, and the Saudi ministry of health [17,43,44]. It has been noted that relying on official and reliable sources for information about pandemics has enhanced the perceptions of HCWs’ about the pandemics and vaccination [15].

HCWs with increased worry about the emergence of the multi-country outbreak of Monkeypox disease and worry of international travel restrictions were predicted to support implementing tighter infection control measures, compared to those measures currently applied. This worry could be further fueled by the recent announcement of the World Health Organization (WHO) that the current outbreak constitutes a public health emergency of international concern (PHEIC) [45]. In addition, HCWs with a family size of >3 persons were significantly predicted to support the application of tighter infection control measures. The family size would heighten alert and create a high burden, as well as increasing the odds of hospitalization; as shown in one study, that the odds in a family of size 4 or more was 2.5 [46].

## 5. Strengths and Limitations

The current study carries the advantage of earlier exploration of HCWs on their perceptions, worries, and vaccination support regarding the evolving Monkeypox disease, within the first month of the WHO alert, and its reemergence in outbreaks in multiple countries outside the African continent. This current alert by the WHO is worth studying, due to the exceptional demand faced by the fatigued international healthcare system, by the resolving COVID-19 pandemic. Still, the present study has limitations related to its survey-based design, such as the convenience sampling technique and recall bias. Another limitation is the scarcity of such cases in the Saudi healthcare system and the unfamiliarity of HCWs with the disease, which may affect their perceptions, worries, and even knowledge. Even so, the current study provides highlights to local healthcare administrators about the HCWs’ perceptions and readiness for Monkeypox, especially in case it progresses into a local or international pandemic.

## 6. Conclusions

The current study has shown that almost half of HCWs within Saudi Arabia are more worried of Monkeypox disease, when compared to COVID-19. Such worries are mainly related to their concern regarding its potential progression into another pandemic. Therefore, the majority were in favor of applying tighter infection prevention measures to combat the disease. The current study highlights areas upon which the healthcare administrative bodies may focus, in order to understand the HCWs’ perceptions about Monkeypox disease and improve their readiness to combat it, both psychologically and medically, in the event that it progresses into a local or international pandemic.

## Figures and Tables

**Table 1:** Participants’ baseline sociodemographic and professional characteristics. N = 1130.

Characteristic	Frequency	Percentage
**Sex**		
Female	708	62.7
Male	422	37.3
**Age group**		
20–30 years	321	28.5
31–40 years	450	39.9
41–50 years	227	20.1
≥51–60 years	129	11.5
**Marital state**		
Never married	381	33.8
Married	746	66.2
**Nationality**		
Saudi	486	43
Expatriate	644	57
**Households (family) size—members**		
Prefer not to answer	122	10.8
1–3 members	446	39.6
4–6 persons	408	36.2
7–10 persons or more	151	13.4
**Households’ monthly income (Saudi Riyals)**		
Prefer not to answer	354	31.4
Less than 10,000 SR	257	22.8
10,001–15,000 SR	118	10.5
15,001–20,000 SR	120	10.6
>20,000 SR	278	24.7
**Working hospital type**		
Primary healthcare center	225	20
Secondary—care hospital	202	17.9
Tertiary hospital	700	62.1
**Clinical Role**		
Medical student	80	7.1
Technicians/Lab workers and Pharmacists	132	11.7
Nurses	470	41.7
Physicians	445	39.5
**Hospital Working area/covering service**		
Pharmacy and laboratory	193	17.1
Critical care units	261	23.1
Infectious Disease/Isolation wards	43	3.8
General wards	297	26.4
OPD	333	29.5
**Have you been previously diagnosed with COVID19?**		
Yes	469	41.6
No	658	58.4

**Table 2:** HCWs’ awareness and sources of information about Monkeypox disease.

Variable	Frequency	Percentage
**Have you travelled in the last month to a country where Monkeypox was recently reported?**		
West or Central Africa	7	5.8
Europe, North America, and Australia	35	52.4
UAE	28	23.3
Other (far Asia, India, Spain, France, and middle eastern countries)	57	47.5
**How would you rate your current awareness about Monkeypox?**		
Low	451	40.1
Moderate	415	36.8
High	261	23.2
**After Receiving this survey, did you perceive the need to read more about Monkeypox disease?**		
No	291	25.8
Yes	836	74.2
**What are your sources of Information about Monkeypox disease?**		
Official local statements or press releases from MOH or Saudi-CDC (Weqayah)	608	57.6
International health authorities’ websites (WHO or CDC)	631	59.8
Social Networks (like YouTube, Facebook, Twitter, and WhatsApp)	539	51.1
Scientific journals	259	24.5
Other Internet-based sources	32	3

**Table 3:** HCW’s Monkeypox disease perceptions and worries.

**How worried are you that Monkeypox will cause a worldwide pandemic similar to COVID-19?**		
None/little worried	549	48.7
Moderate worry	293	26
Worried a lot	285	25.3
**Do you think Monkeypox causes a more severe disease compared to Smallpox**?		
Disagree	264	23.4
Unsure	448	39.8
Agree	415	36.8
**Which is more worrisome to you, COVID-19 or Monkeypox disease?**		
Unsure/Equally worried	88	7.8
I am more worried about COVID-19	637	56.5
I am more worried about Monkeypox	402	35.7
**Healthcare workers should apply more infection control measures than the current ones, with the new Monkeypox outbreaks in some countries?**		
Agree	762	67.6
Neither agree nor disagree	292	25.9
Disagree	73	6.5
**Please rate your level of worry about traveling abroad with the new Monkeypox outbreaks in some countries**		
Not worried at all	230	20.4
Somewhat worried	745	66.1
Extremely worried	152	13.5
**HCWs’ sources of worries from Monkeypox disease**		
Me or my family being affected by the Monkeypox	395	49.6
Another worldwide pandemic	548	68.8
Worried Monkeypox might surge to cause national lock-down	339	42.6
International flight suspension	325	40.8
Other (please specify)	32	4

**Table 4:** Multivariate binary logistic regression analysis of the HCW’s odds of high worry from Monkeypox compared to COVID-19.

	Multivariate Adjusted Odds Ratio (OR)	95% CI		*p* Value
		Lower	Upper	
Sex = Male	0.533	0.394	0.721	<0.001
Age (years)	1.000	0.984	1.017	0.964
Previous COVID-19 infection	0.735	0.560	0.964	0.026
Households Size-Family size	1.115	0.945	1.314	0.196
Clinical Role Medical student	2.796	1.570	4.978	<0.001
Self-rated awareness of Monkeypox disease score	0.765	0.661	0.885	<0.001
Worry from Monkeypox progression into a pandemic	1.465	1.275	1.682	<0.001
Perception of Monkeypox as a severe disease	1.263	1.043	1.529	0.017
GAD7 score	0.987	0.958	1.017	0.393
Agreement with the application of tighter infection control measures	1.691	1.294	2.210	<0.001
High worry from international travel restrictions	1.206	0.934	1.557	0.151
Constant	0.056			<0.001

DV = High worry from Monkeypox disease.

**Table 5:** Multivariate binary logistic regression analysis of the HCW’s odds of supporting vaccinations against Monkeypox disease.

	Multivariate Adjusted Odds Ratio (OR)	95% CI		*p*-Value
		Lower	Upper	
Sex Male	0.899	0.670	1.206	0.478
Age (years)	0.993	0.976	1.010	0.416
Marital state Ever married	0.891	0.629	1.261	0.515
Type of institution Tertiary	1.232	0.934	1.624	0.140
Previous COVID-19 infection	1.327	1.009	1.745	0.043
Agreement with the application of tighter infection control measures	1.415	1.125	1.781	0.003
HHI	1.019	0.931	1.116	0.682
Perception of need to read more about Monkeypox	1.248	0.896	1.737	0.189
GAD7	1.006	0.977	1.037	0.673
Source of information				
Local official statements	2.023	1.515	2.702	<0.001
International health	1.375	1.031	1.833	0.030
Social media channels	1.953	1.468	2.598	<0.001
Constant	0.054			<0.001

DV = HCW’s odds of supporting vaccinations against Monkeypox disease.

**Table 6:** Multivariate binary logistic regression analysis of the HCW’s odds of supporting the Implementation of tighter infection control measures against Monkeypox compared to those currently applied.

	Multivariate Adjusted Odds Ratio (OR)	95% CI		*p*-Value
		Lower	Upper	
Sex Male	0.863	0.631	1.179	0.354
Age (years)	0.982	0.964	1.001	0.060
Ever married	1.365	0.936	1.991	0.106
Clinical role	0.891	0.743	1.069	0.213
Perception to read more about Monkeypox	3.157	2.268	4.393	<0.001
GAD7	1.012	0.978	1.048	0.490
Source of information				
International health websites	1.242	0.900	1.712	0.187
Social media/networks channels	1.353	1.005	1.820	0.046
Science journals	0.656	0.456	0.943	0.023
More worried about Monkeypox compared to COVID-19	2.067	1.469	2.908	<0.001
Worry level from international travel restrictions	1.568	1.184	2.078	0.002
Household size ≥ 4 persons	1.230	1.031	1.466	0.021
Worry Monkeypox progresses into a pandemic	0.813	0.703	0.941	0.006
Perception of Monkeypox as a severe disease	1.316	1.072	1.617	0.009
Constant	0.120			0.002

DV = Supporting implementation of tighter infection control measures against Monkeypox compared to those currently applied.

## Data Availability

Available upon reasonable request from the corresponding author.

## Abbreviations

CDC	Centers for Disease Control and Prevention
COVID-19	Coronavirus disease 2019
MOH	Ministry of Health
MPXV	Monkeypox virus
SARS-CoV-2	Severe acute respiratory syndrome coronavirus 2
WHO	World Health Organization

## References

[1] Al-Tawfiq J.A., Barry M., Memish Z.A. (2022). International outbreaks of Monkeypox virus infection with no established travel: A public health concern with significant knowledge gap. Travel Med. Infect. Dis..

[2] León-Figueroa D.A., Bonilla-Aldana D.K., Pachar M., Romaní L., Saldaña-Cumpa H.M., Anchay-Zuloeta C., Diaz-Torres M., Franco-Paredes C., Suárez J.A., Ramirez J.D. (2022). The never-ending global emergence of viral zoonoses after COVID-19? The rising concern of monkeypox in Europe, North America and beyond. Travel Med. Infect. Dis..

[3] Kozlov M. (2022). Monkeypox goes global: Why scientists are on alert. Nature.

[4] Meeting of the International Health Regulations (2005) Emergency Committee Regarding the Multi-Country Monkeypox Outbreak. https://www.who.int/news/item/25-06-2022-meeting-of-the-international-health-regulations-(2005)-emergency-committee--regarding-the-multi-country-monkeypox-outbreak.

[5] Temsah M.H., Aljamaan F., Alenezi S., Alhasan K., Saddik B., Al-Barag A., Alhaboob A., Bahabri N., Alshahrani F., Alrabiaah A. (2022). Monkeypox caused less worry than COVID-19 among the general population during the first month of the WHO Monkeypox alert: Experience from Saudi Arabia. Travel Med. Infect. Dis..

[6] Al-Tawfiq J.A., Temsah M.H. (2022). Perspective on the challenges of COVID-19 facing healthcare workers. Infection.

[7] Kozlov M. (2022). Monkeypox vaccination begins-can the global outbreaks be contained?. Nature.

[8] Selb R., Werber D., Falkenhorst G., Steffen G., Lachmann R., Ruscher C., McFarland S., Bartel A., Hemmers L., Koppe U. (2022). A shift from travel-associated cases to autochthonous transmission with Berlin as epicentre of the monkeypox outbreak in Germany, May to June. Eurosurveillance.

[9] Iñigo Martínez J., Gil Montalbán E., Jiménez Bueno S., Martín Martínez F., Nieto Juliá A., Sánchez Díaz J., García Marín N., Córdoba Deorador E., Nunziata Forte A., Alonso García M. (2022). Monkeypox outbreak predominantly affecting men who have sex with men, Madrid, Spain, 26 April to 16 June. Eurosurveillance.

[10] McAndrew T., Majumder M.S., Lover A.A., Venkatramanan S., Bocchini P., Besiroglu T., Codi A., Braun D., Dempsey G., Abbott S. (2022). Early human judgment forecasts of human monkeypox, May 2022. Lancet Digit. Health.

[11] World Health Organization (WHO) (2022). Vaccines and Immunization for Monkeypox: Interim Guidance, 14 June 2022.

[12] Simpson L.A., Macdonald K., Searle E.F., Shearer J.A., Dimitrov D., Foley D., Morales E., Shenoy E.S. (2022). Development and deployment of tools for rapid response notification of Monkeypox exposure, exposure risk assessment and stratification, and symptom monitoring. Infect. Control Hosp. Epidemiol..

[13] Temsah M.H., Alhuzaimi A.N., Alamro N., Alrabiaah A., Al-Sohime F., Alhasan K., Kari J.A., Almaghlouth I., Aljamaan F., Al Amri M. (2020). Knowledge, Attitudes, and Practices of Healthcare Workers during the Early COVID-19 Pandemic in a Main, Academic Tertiary Care Centre in Saudi Arabia. Epidemiol. Infect..

[14] Temsah M.H., Al-Sohime F., Alamro N., Al-Eyadhy A., Al-Hasan K., Jamal A., Al-Maglouth I., Aljamaan F., Al Amri M., Barry M. (2020). The psychological impact of COVID-19 pandemic on health care workers in a MERS-CoV endemic country. J. Infect. Public Health.

[15] Temsah M.H., Barry M., Aljamaan F., Alhuzaimi A.N., Al-Eyadhy A., Saddik B., Alsohime F., Alhaboob A., Alhasan K., Alaraj A. (2021). SARS-CoV-2 B.1.1.7 UK Variant of Concern Lineage-Related Perceptions, COVID-19 Vaccine Acceptance and Travel Worry among Healthcare Workers. Front. Public Health.

[16] Barry M., Temsah M.-H., Aljamaan F., Saddik B., Al-Eyadhy A., Alanazi S., Alamro N., Alhuzaimi A., Alhaboob A., Alsohime F. (2021). COVID-19 vaccine uptake among healthcare workers in the fourth country to authorize BNT162b2 during the first month of rollout. Vaccine.

[17] Alhasan K., Aljamaan F., Temsah M.H., Alshahrani F., Bassrawi R., Alhaboob A., Assiri R., Alenezi S., Alaraj A., Alhomoudi R.I. (2021). COVID-19 Delta Variant: Perceptions, Worries, and Vaccine-Booster Acceptability among Healthcare Workers. Healthcare.

[18] Spitzer R.L., Kroenke K., Williams J.B., Lowe B. (2006). A brief measure for assessing generalized anxiety disorder: The GAD-7. Arch. Intern. Med..

[19] AlHadi A.N., AlAteeq D.A., Al-Sharif E., Bawazeer H.M., Alanazi H., AlShomrani A.T., Shuqdar R.M., AlOwaybil R. (2017). An arabic translation, reliability, and validation of Patient Health Questionnaire in a Saudi sample. Ann. Gen. Psychiatry.

[20] Monkeypox. https://www.who.int/news-room/fact-sheets/detail/monkeypox.

[21] Petersen E., Abubakar I., Ihekweazu C., Heymann D., Ntoumi F., Blumberg L., Asogun D., Mukonka V., Lule S.A., Bates M. (2019). Monkeypox-Enhancing public health preparedness for an emerging lethal human zoonotic epidemic threat in the wake of the smallpox post-eradication era. Int. J. Infect. Dis..

[22] Harapan H., Setiawan A.M., Yufika A., Anwar S., Wahyuni S., Asrizal F.W., Sufri M.R., Putra R.P., Wijayanti N.P., Salwiyadi S. (2020). Confidence in managing human monkeypox cases in Asia: A cross-sectional survey among general practitioners in Indonesia. Acta Trop..

[23] Harapan H., Setiawan A.M., Yufika A., Anwar S., Wahyuni S., Asrizal F.W., Sufri M.R., Putra R.P., Wijayanti N.P., Salwiyadi S. (2020). Knowledge of human monkeypox viral infection among general practitioners: A cross-sectional study in Indonesia. Pathog. Glob. Health.

[24] Harapan H., Wagner A.L., Yufika A., Setiawan A.M., Anwar S., Wahyuni S., Asrizal F.W., Sufri M.R., Putra R.P., Wijayanti N.P. (2020). Acceptance and willingness to pay for a hypothetical vaccine against monkeypox viral infection among frontline physicians: A cross-sectional study in Indonesia. Vaccine.

[25] Graham F. (2022). Daily briefing: Why scientists are worried about monkeypox. Nature.

[26] Spoorthy M.S., Pratapa S.K., Mahant S. (2020). Mental health problems faced by healthcare workers due to the COVID-19 pandemic-A review. Asian J. Psychiatr..

[27] Huang Q., Luo L.S., Wang Y.Y., Jin Y.H., Zeng X.T. (2021). Gender Differences in Psychological and Behavioral Responses of Infected and Uninfected Health-Care Workers during the Early COVID-19 Outbreak. Front. Public Health.

[28] Liu S., Yang L., Zhang C., Xu Y., Cai L., Ma S., Wang Y., Cai Z., Du H., Li R. (2021). Gender differences in mental health problems of healthcare workers during the coronavirus disease 2019 outbreak. J. Psychiatr. Res..

[29] Gupta P., BK A., Ramakrishna K. (2021). Prevalence of Depression and Anxiety among Medical Students and House Staff during the COVID-19 Health-Care Crisis. Acad. Psychiatry.

[30] Breugnon E., Thollot H., Fraissenon A., Saunier F., Labetoulle R., Pillet S., Lucht F., Berthelot P., Botelho-Nevers E., Gagneux-Brunon A. (2021). COVID-19 outpatient management: Shorter time to recovery in Healthcare workers according to an electronic daily symptoms assessment. Infect. Dis. Now.

[31] Trumello C., Bramanti S.M., Ballarotto G., Candelori C., Cerniglia L., Cimino S., Crudele M., Lombardi L., Pignataro S., Viceconti M.L. (2020). Psychological Adjustment of Healthcare Workers in Italy during the COVID-19 Pandemic: Differences in Stress, Anxiety, Depression, Burnout, Secondary Trauma, and Compassion Satisfaction between Frontline and Non-Frontline Professionals. Int. J. Environ. Res. Public Health.

[32] Buselli R., Corsi M., Baldanzi S., Chiumiento M., Del Lupo E., Dell’Oste V., Bertelloni C.A., Massimetti G., Dell’Osso L., Cristaudo A. (2020). Professional Quality of Life and Mental Health Outcomes among Health Care Workers Exposed to SARS-CoV-2 (COVID-19). Int. J. Environ. Res. Public Health.

[33] Moccia L., Janiri D., Pepe M., Dattoli L., Molinaro M., De Martin V., Chieffo D., Janiri L., Fiorillo A., Sani G. (2020). Affective temperament, attachment style, and the psychological impact of the COVID-19 outbreak: An early report on the Italian general population. Brain Behav. Immun..

[34] Loveday H. (2020). Fear, explanation and action—The psychosocial response to emerging infections. J. Infect. Prev..

[35] Russo A.T., Berhanu A., Bigger C.B., Prigge J., Silvera P.M., Grosenbach D.W., Hruby D. (2020). Co-administration of tecovirimat and ACAM2000™ in non-human primates: Effect of tecovirimat treatment on ACAM2000 immunogenicity and efficacy versus lethal monkeypox virus challenge. Vaccine.

[36] Costello V., Sowash M., Gaur A., Cardis M., Pasieka H., Wortmann G., Ramdeen S. (2022). Imported Monkeypox from International Traveler, Maryland, USA, 2021. Emerg Infect Dis.

[37] Barry M., Temsah M.H., Alhuzaimi A., Alamro N., Al-Eyadhy A., Aljamaan F., Saddik B., Alhaboob A., Alsohime F., Alhasan K. (2021). COVID-19 vaccine confidence and hesitancy among health care workers: A cross-sectional survey from a MERS-CoV experienced nation. PLoS ONE.

[38] Bertoni L., Roncadori A., Gentili N., Danesi V., Massa I., Nanni O., Altini M., Gabutti G., Montella M.T. (2022). How has COVID-19 pandemic changed flu vaccination attitudes among an Italian cancer center healthcare workers?. Hum. Vaccines Immunother..

[39] Pérez-Rivas F.J., Gallego-Lastra R.D., Marques-Vieira C.M.A., López-López C., Domínguez-Fernández S., Rico-Blázquez M., Ajejas Bazán M.J. (2022). The Attitude towards Vaccination of Health Sciences Students at a Spanish University Improved over the First 18 Months of the COVID-19 Pandemic. Vaccines.

[40] Akibu M., Nurgi S., Tadese M., Tsega W.D. (2018). Attitude and Vaccination Status of Healthcare Workers against Hepatitis B Infection in a Teaching Hospital, Ethiopia. Scientifica.

[41] Rao A.K., Petersen B.W., Whitehill F., Razeq J.H., Isaacs S.N., Merchlinsky M.J., Campos-Outcalt D., Morgan R.L., Damon I., Sánchez P.J. (2022). Use of JYNNEOS (Smallpox and Monkeypox Vaccine, Live, Nonreplicating) for Preexposure Vaccination of Persons at Risk for Occupational Exposure to Orthopoxviruses: Recommendations of the Advisory Committee on Immunization Practices—United States, 2022. Morb. Mortal. Wkly. Rep..

[42] Temsah M.H., Aljamaan F., Alenezi S., Alhasan K., Alrabiaah A., Assiri R., Bassrawi R., Alhaboob A., Alshahrani F., Alarabi M. (2022). SARS-CoV-2 Omicron Variant: Exploring Healthcare Workers’ Awareness and Perception of Vaccine Effectiveness: A National Survey During the First Week of WHO Variant Alert. Front. Public Health.

[43] Temsah M.H., Barry M., Aljamaan F., Alhuzaimi A., Al-Eyadhy A., Saddik B., Alrabiaah A., Alsohime F., Alhaboob A., Alhasan K. (2021). Adenovirus and RNA-based COVID-19 vaccines’ perceptions and acceptance among healthcare workers in Saudi Arabia: A national survey. BMJ Open.

[44] Temsah M.H., Alenezi S., Alarabi M., Aljamaan F., Alhasan K., Assiri R., Bassrawi R., Alshahrani F., Alhaboob A., Alaraj A. (2022). Healthcare Workers’ SARS-CoV-2 Omicron Variant Uncertainty-Related Stress, Resilience, and Coping Strategies during the First Week of the World Health Organization’s Alert. Int. J. Environ. Res. Public Health.

[45] Second Meeting of the International Health Regulations (IHR) (2005). Emergency Committee Regarding the Multi-Country Outbreak of Monkeypox. https://www.who.int/news/item/23-07-2022-second-meeting-of-the-international-health-regulations-(2005)-(ihr)-emergency-committee-regarding-the-multi-country-outbreak-of-monkeypox.

[46] Nash D., Qasmieh S., Robertson M., Rane M., Zimba R., Kulkarni S.G., Berry A., You W., Mirzayi C., Westmoreland D. (2022). Household factors and the risk of severe COVID-like illness early in the U.S. pandemic. PLoS ONE.

